# Prevalence of Klebsiella pneumoniae Antibiotic Resistance in Medina, Saudi Arabia, 2014-2018

**DOI:** 10.7759/cureus.9714

**Published:** 2020-08-13

**Authors:** Abdulmohsen Al-Zalabani, Oussama A AlThobyane, Ayoub H Alshehri, Abdulaziz O Alrehaili, Mohammad O Namankani, Owais H Aljafri

**Affiliations:** 1 Family Medicine, Taibah University, Medina, SAU; 2 Medicine, Taibah University, Medina, SAU; 3 Medicine, Taibah University, Madina, SAU

**Keywords:** klebsiella pneumoniae, antibiotic resistance, esbl, saudi arabia, medina, multi-drug resistance, cross-resistance

## Abstract

Antibiotic resistance is a rising dilemma of significant implications on global public health. Few data exist for the prevalence and trends of *Klebsiella pneumonia* antibiotic resistance in Saudi Arabia. Therefore, we have set out to identify the prevalence and trends of *Klebsiella pneumonia* antibiotic resistance in King Fahad Hospital in Medina over the period between February 27, 2014, and December 31, 2018. The research was carried out as a retrospective cross-sectional study. A total of 15708 isolates taken from 1149 patients were included in the study. Statistical analysis was done using SPSS (IBM Corp, Armonk, NY). We used descriptive and trend analysis using the linear regression method. In the results, we have found unprecedented emergence of resistance to carbapenems, with 38.4% (n=436) for imipenem and 46.1% (n=371) for meropenem, which are the first choice of treatment in local guidelines, as well as high resistance rates for commonly used alternative options of treatment (40.7% (n=105) for colistin and 53.3% (n=220) for tigecycline). In addition, third and fourth generation cephalosporins showed resistance ranging from 57.5% to 77.8%. Co-resistance with imipenem was found at rates exceeding 75% for other choices of management (aminoglycosides and cephalosporins), apart from colistin and tigecycline that had rates of 53.6% (n=89) and 61.4% (n=167), respectively. In conclusion, the research showed increased resistance rates to beta-lactams, as well as an emergence of resistance to carbapenems and other alternatives of treatment like colistin and tigecycline.

## Introduction

Antibiotic therapy is one of the great advances of the 20th century that has succeeded in preventing a great number of infectious diseases and premature deaths. In both the developing and industrialized world, infections like smallpox, typhoid, plague, cholera, etc. were rampant, and the average life expectancy was significantly lower [1]. In the US, for example, it changed from 47.3 years in the pre-World War I era to 68.2 years in 1950, and the leading cause of mortality changed from communicable to non-communicable diseases [2]. However, antibiotic resistance is a rising dilemma of significant implications on global public health, showcased by increasing levels of morbidity and mortality, as well as a strain on health care systems [3]. Antibiotic misuse is regarded as the main contributor to resistance [4], and the magnitude of the problem in the Kingdom of Saudi Arabia (KSA) is showcased by a prevalence of misuse ranging between 41% and 92% [5]. Surveillance of antibiotic susceptibility plays an important role in monitoring the emergence of resistance and guiding interventions [3,6].

*Klebsiella (K.) pneumoniae* is a gram-negative, facultative anaerobic, non-motile, and non-flagellated bacillus that is a member of the Enterobacteriaceae family. It is found in the respiratory tract and stools of about 5% of normal individuals. It is responsible for about 1% of bacterial pneumonia. *Klebsiella* species rank among the top 10 bacteria causing hospital-acquired infections, and it is one of the most common pathogens isolated in the intensive care unit (ICU) [7-9]. Among these infections, *K. pneumoniae* is responsible for a significant proportion of urinary tract infections, pneumonia, and soft tissue infections. The principal ways of transmission of Klebsiella are through the gastrointestinal tract and hands of hospital staff [8].

The increased use of antimicrobial agents and continuous exposure of*K. pneumoniae* to them has resulted in the emergence of multi-drug resistant *K. pneumoniae *[10]. Outbreaks of multidrug-resistant (MDR)*K. pneumoniae* in hospitals is attributed to their ability to spread rapidly. Outbreaks are often caused by new types of *Klebsiella* strains; these strains are called extended-spectrum-β-lactamase (ESBL) producers. The incidence of ESBL producers has been increasing steadily in the past years, causing limitations in therapeutic options [11-12]. Most importantly, the emergence of carbapenem-resistant *Klebsiella pneumoniae* is a global health concern and is listed by the World Health Organization (WHO) as a critical priority [13]. Besides that, the presence of ESBL-producing strain has been reported in Saudi Arabia [14].

The clinical significance of the pathogen and its implications on infection control, as well as the emergence of new resistant strains, all signify the importance of local surveillance of antibiotic-resistant organisms and tracking emerging resistance. And there are few data regarding the prevalence and trends of *K. pneumoniae* in Saudi Arabia as a whole and the Medina region in particular [15-17]. This is why we aim to establish the prevalence of antimicrobial drug resistance and the susceptibility of *Klebsiella pneumoniae* in King Fahad Hospital (KFH) in Medina in the previous five years, starting from January 1, 2014, to December 31, 2018.

## Materials and methods

Study design and setting

We have conducted this research as a cross-sectional study to assess the prevalence of antibiotic-resistant *K. pneumoniae*. The data was gathered from King Fahad Hospital, Medina, Saudi Arabia, in the duration between January 1, 2014, and December 31, 2018, and in that period, the number of samples collected was 27384 isolates. Taken from a total of 1149 patients. *K. pneumoniae* isolates were identified from the records of the microbiology lab in KFH where antibiotic testing susceptibility was carried out using an automated system (VITEK 2 system, BioMérieux, Marcy-l'Étoile, France), following the Clinical and Laboratory Standards Institute (CLSI) interpretation guidelines: 24th Edition, 2014.

Inclusion/exclusion criteria

We included samples collected from inpatients and only included the first samples taken from each patient. Samples were collected from various bodily fluids. We excluded any secondary cultures of the same patient, so as to avoid repeated isolates. We also excluded outpatient samples. Secondary cultures were identified as a sample taken within six months of the first sample.

Statistical analysis

Our main objective of this study was to assess the prevalence of *K. pneumoniae *resistance to antibiotics over the duration of the study. Statistical analysis was performed using the Statistical Package for the Social Sciences (SPSS Version 23, IBM Corp, Armonk, NY). Descriptive analysis was done using cross tabs. Data were expressed as a number and percentage. Trend analysis was done using the linear regression method. Furthermore, the regression coefficient was calculated and included only if it was significant, P-value < 0.05.

## Results

A total of 27384 results of *K. pneumoniae* antimicrobial resistance were identified from a total of 1149 inpatients within the study period. A total of 11676 (42.63%) were secondary samples (repeated within six months of the first), and they were excluded in accordance with our criteria. The remaining samples (first samples), which were 15708 (57.73%), were subjected to further analysis.

We found high resistance rates in commonly used antibiotics like amoxicillin/clavulanic acid at 72% (n=572) in total, and it showed an increase between 2015 (71%) and 2017 (75.8%). Ampicillin showed a resistance rate of 99.9% (n=685) where only one sample was sensitive to the drug. Piperacillin showed a high resistance rate as well at 80.4% (n=288). A piperacillin/tazobactam combination, however, had lower resistance at 58.7% (n=505). Another combination (trimethoprim/sulfamethoxazole) showed high resistance at 67% (n=693).

Fluoroquinolone showed lower resistance rates, with levofloxacin being the lowest of the class with 57.7% (n=384) and ciprofloxacin at 61.1% (n=686). Aminoglycosides also showed lower resistance rates, especially with amikacin at 36.3% (n=408); its rate, however, increased from 28.9% (n=54) in 2015 to 39.7% (n=139) in 2018. Gentamicin had a total resistance rate of 52.2% (n=543) and showed a similar increase in rate between 2015 and 2018: 46.1% (n=83) and 52.2% (n=179). Aztreonam (monobactam) showed a resistance rate of 66.3% (n=627) with no apparent changes in rates over the years of the study.

First-generation cephalosporins showed high resistance rates with cephalexin at 92% (n=23), cephalothin at 80.8% (n=563), and cefazolin at 78% (n=71). Second-generation cephalosporins were similar as well with cefuroxime at 73.8% (n=45). Third-generation cephalosporins ranged between 57.5% and 77.8%. Cefoxitin had a resistance rate of 57.5% (n=413), ceftazidime had 66.9% (n=743), cefotaxime with 77% (n=77), and ceftriaxone 77.8% (70). Finally, cefepime (fourth-generation cephalosporin) had 68.4% (n=564) resistance.

With the emergence of extended-spectrum-β-lactamase (ESBL) producing strains and high resistance rates of beta-lactams, carbapenems have become the treatment of choice for ESBL-producing organisms [18]. During our study period, carbapenems showed one of the lower resistance rates with imipenem having a rate of 38.4% (n=436) and had an increase between 2015 and 2018 from 34.6% (n=65) to 38.7% (n=135). Meropenem had a resistance rate of 46.1% (n=371).

**Figure 1:**
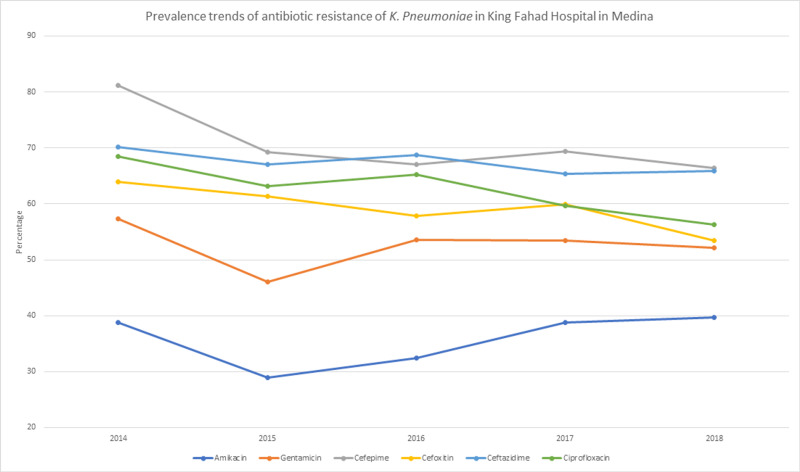
Prevalence trends of the antibiotic resistance of K. pneumoniae in King Fahad Hospital in Medina

**Figure 2:**
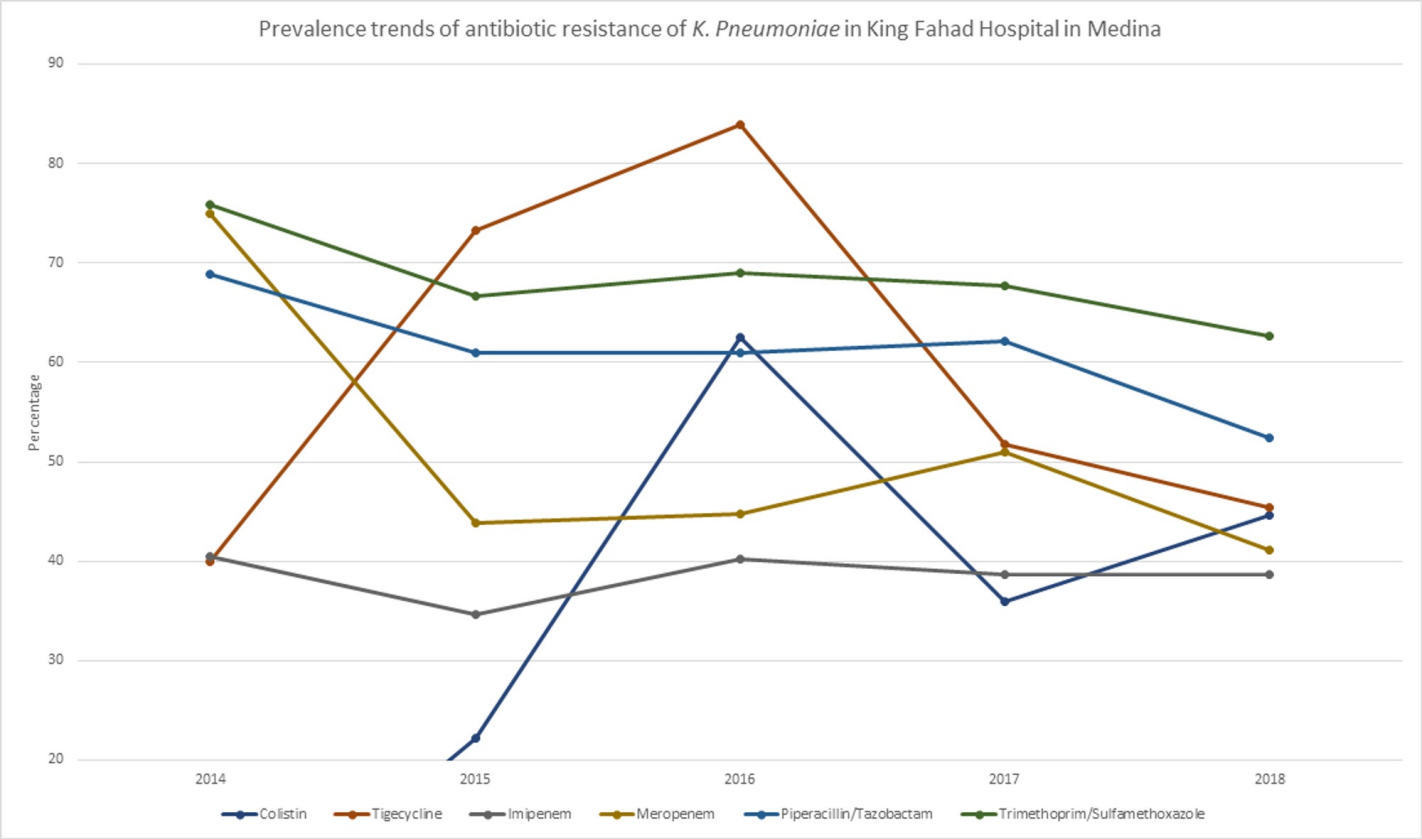
Prevalence trends of the antibiotic resistance of K. pneumoniae in King Fahad Hospital in Medina

Non-beta lactamase antibiotics that have come to be a treatment of choice following the emergence of ESBL and carbapenemase-producing strains of *K. pneumonia* [19] showed worrying resistance rates as well, with colistin at 40.7% (n=105) and presenting an increase of 8.6% between 2017 and 2018. And it’s worth noting that the sample size in the preceding years was very small (>10 samples a year). Tigecycline showed a resistance rate of 53.3% (n=220). In Table 1, we can see the resistance rates of the more clinically used antibiotics. And Figure 1 and Figure 2 show their yearly linear trends throughout the research period.

**Table 1:** K. Pneumoniae antibiotic sensitivity between 2014 and 2018 in King Fahad Hospital in Medina

K. Pneumoniae antibiotic sensitivity between 2014 and 2018 in King Fahad Hospital in Medina														
Table 1		Years												
		2014		2015		2016		2017		2018		Total years		
		R	Total	R	Total	R	Total	R	Total	R	Total	R	Total	
Amikacin	Count	47	121	54	187	64	197	104	268	139	350	408	1123	
	% within Antibiotic	38.8%		28.9%		32.5%		38.8%		39.7%		36.3%		
Gentamicin	Count	63	110	83	180	96	179	122	228	179	343	543	1040	
	% within Antibiotic	57.3%		46.1%		53.6%		53.5%		52.2%		52.2%		
Cefepime	Count	39	48	83	120	94	140	127	183	221	333	564	824	
	% within Antibiotic	81.3%		69.2%		67.1%		69.4%		66.4%		68.4%		
Cefoxitin	Count	53	83	54	88	62	107	85	142	159	298	413	718	
	% within Antibiotic	63.9%		61.4%		57.9%		59.9%		53.4%		57.5%		
Ceftazidime	Count	87	124	122	182	136	198	174	266	224	340	743	1110	
	% within Antibiotic	70.2%		67.0%		68.7%		65.4%		65.9%		66.9%		
Ciprofloxacin	Count	85	124	117	185	129	198	158	265	197	350	686	1122	
	% within Antibiotic	68.5%		63.2%		65.2%		59.6%		56.3%		61.1%		
Colistin	Count	0	4	2	9	5	8	32	89	66	148	105	258	
	% within Antibiotic	0.0%		22.2%		62.5%		36.0%		44.6%		40.7%		
Tigecycline	Count	4	10	11	15	47	56	59	114	99	218	220	414	
	% within Antibiotic	40.0%		73.3%		83.9%		51.8%		45.4%		53.3%		
Imipenem	Count	53	131	65	188	80	199	103	267	135	349	436	1134	
	% within Antibiotic	40.5%		34.6%		40.2%		38.6%		38.7%		38.4%		
Meropenem	Count	27	36	43	98	65	145	101	198	135	327	371	804	
	% within Antibiotic	75.0%		43.9%		44.8%		51.0%		41.1%		46.1%		
Piperacillin/Tazobactam	Count	51	74	83	136	78	128	118	190	174	332	505	861	
	% within Antibiotic	68.9%		61.0%		60.9%		62.1%		52.4%		58.7%		
Trimethoprim/sulfamethoxazole	Count	88	116	90	135	120	174	180	266	214	342	693	1034	
	% within Antibiotic	75.9%		66.7%		69.0%		67.7%		62.6%		67.0%		
Total of tested antibiotics	Count	1009	1473	1232	2100	1562	2511	2521	3948	3368	5676	9692	15708	
	% within Antibiotic	68.5%		58.7%		62.2%		63.9%		59.3%		61.7%		

**Figure 3:**
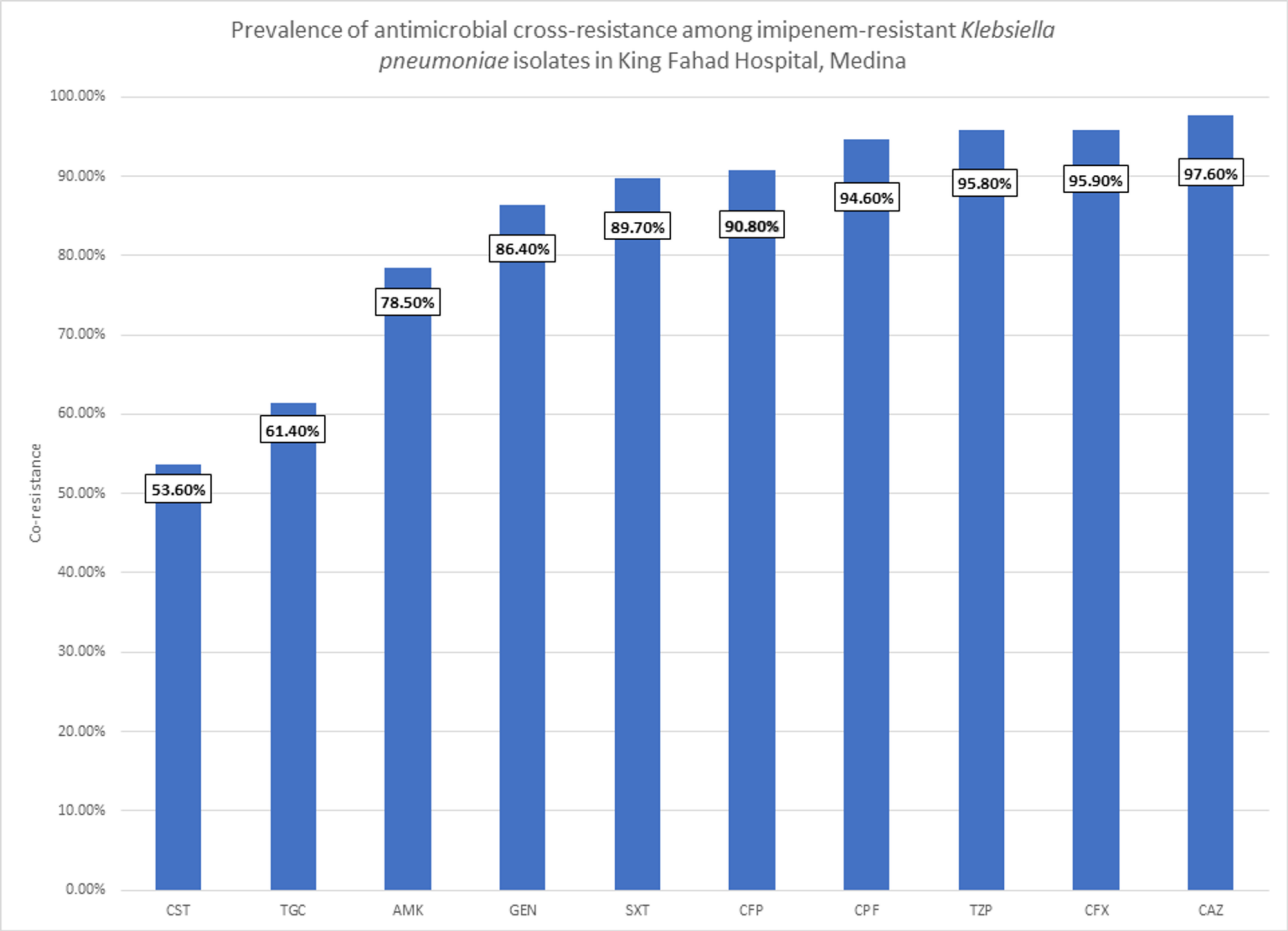
Prevalence of antimicrobial cross-resistance among imipenem-resistant Klebsiella pneumoniae isolates in King Fahad Hospital, Medina CST: Colistin, TGC: Tigecycline, AMK: Amikacin, GEN: Gentamycin, SXT: Trimethoprim/Sulfamethoxazole, CFP: Cefepime, CPF: Ciprofloxacin, TZP: Piperacillin/Tazobactam, CFX: Ceftriaxone, CAZ: Ceftazidime

As imipenem is the first choice in managing *K. pneumonia* [20], we set out to assess the cross-resistance of imipenem with other antibiotics, in order to compare the available options of treatment following imipenem-resistance. In Figure 3, the prevalence of antimicrobial cross-resistance among imipenem-resistant *Klebsiella pneumoniae* isolates is shown.

We can see cephalosporins all being highly resistant as expected in patients with carbapenem resistance, with cefepime having the lowest resistance at 90.8% (n=334). Amikacin and gentamicin were a better option at 78.5% (n=333) and 86.4% (n=361), respectively. Trimethoprim/sulfamethoxazole combination showed a high resistance rate of 89.7% (n=350). Piperacillin/tazobactam was at 95.8% (n-364).

Now, the antibiotics with the highest sensitivity were non-beta-lactam antibiotics that have come to be an option of treatment with the resistance of beta-lactams and carbapenems [19]. Colistin had a resistance rate of 53.6% (n=89). In addition, tigecycline was at 61.4% (n=167).

## Discussion

As we look at guidelines for *K. pneumoniae* treatment, recommendations include using third-generation cephalosporins (e.g. cefotaxime, ceftriaxone) as first-line management for non-resistant organisms [21]. However, after the analysis of obtained samples from KFH over the four-year duration of the study, we have found that resistant patterns for this group in our study were alarmingly high, at 68.4% (n=564) and 66.9% (n=743) for cefepime and ceftazidime, respectively.

Although the emergence of resistance for beta-lactams has been noted in previous research [22], we have set out to identify the emergence of antimicrobial resistance in both beta-lactams, as well as options of treatment following the emergence of ESBL-producing strains. The last updated guidelines for the Ministry of Health (MOH) in Saudi Arabia put carbapenems as the first choice of treatment, which means that identifying its resistance is of paramount importance [20].

Our main objective of this study was to assess the prevalence of *K. pneumoniae* resistance to antibiotics over the duration of the study. As well as present trends of its resistance over the years. The overall resistance found in 11507 isolates was 61.7 %, with a significant increase over the years 2015-2018 at a regression coefficient of +0.233.

Among carbapenems, which are the first choice of treatment in Saudi guidelines, the levels of resistance for imipenem was 38.4% (n=436), and for meropenem 46.1% (n=471). This is worrying, as this group of antibiotics is considered the choice of treatment against ESBL-producing *K. pneumoniae.* This resistance rate signifies the possible emergence of carbapenemase-producing strains.

Moreover, amikacin was the most effective antibiotic against *K. pneumoniae* at 36.3% (n=408) resistance; unfortunately, it showed a significant increase over the years 2015-2018 at a regression coefficient of +0.103. Although the year 2014 had a low sample size, which is why it was excluded from the analysis.

Looking at non-beta-lactam antibiotics that have come to be an option of treatment following the emergence of carbapenem-resistant *K. pneumoniae* [19]. Colistin was surprisingly potent against *K. pneumoniae* at 40.7% resistance. Nevertheless, due to low overall samples at 258 isolates, of which only 21 samples were done in the years 2014-2016, the results were unreliable. Although the result remains a worry because of limited choices of treatment, tigecycline showed a resistance rate of 53.3% (n=220).

Regional antibiotic-resistance patterns help in establishing guidelines over clinical choices in managing these infections. Few studies regarding *K. pneumoniae *antibiotic resistance were done in recent years, especially in Saudi Arabia. A study by Al-Tawfik et al. was published in the year 2007, in which they studied the trends of antibiotic resistance in *K. pneumonia*e from 1998-2003 [17]. In the study, an increasing rate of resistance against three specific antibiotics was found. An important finding to notice in the mentioned study was that imipenem had virtually no resistance in all 204 samples from outpatients as well as 218 samples of hospital-acquired *K. pneumoniae* that were collected and tested for imipenem sensitivity. On the contrary, we found in our research that imipenem sensitivity samples had a significantly higher resistance rate at 38.4% (n=436), this result might point towards the emergence of carbapenemase-producing strains. Carbapenem resistance has been reported in southern Saudi Arabia in 2018, although there were no noted resistance rates in patient populations [14].

With carbapenems as one of the first choices of treatment for *K. pneumoniae* [20], we analyzed the antibiotics that have cross-resistance with imipenem to evaluate treatment options should carbapenems be resistant. We have found that the antibiotics with the least resistance were colistin at 53.6% (n=89) and tigecycline at 61.4% (n=167).

Antibiotic misuse is a crucial problem in Saudi Arabia and is signified by a prevalence of misuse, ranging between 41% and 92% [5]. A study done in 2016 in 26 Saudi hospitals showed that third-generation cephalosporins are the most commonly prescribed antibiotics [5]. And due to the link between broad-spectrum cephalosporin use and the emergence of ESBL and carbapenemase-producing strains [23], which explain antimicrobial resistance, we can theorize a link between the high resistance rates found in our study and antibiotic misuse in the region.

The limitations of our method include the lack of strain testing in the data, which could differentiate ESBL, carbapenemase-producing strains, and MDR *K. pneumoniae*. Another limitation was the inability to generalize the results to the Medina region because other hospitals in the region did not have either the equipment or enough data to be included in our study. However, KFH remains the biggest in the region. Moreover, the system used in identifying antibiotic sensitivity was adopted by KFH in early 2014, therefore, the samples collected in 2014 were significantly smaller than the following years and no samples were recorded in earlier years.

The strength of our study was the sample size; we analyzed 15708 samples from 1149 patients. A number of this magnitude gives our study credibility and statistical significance. Another strength of our research is its relevance to the current local climate. In April 2018, the Ministry of Health in Saudi Arabia established a decision to limit the usage of non-prescription antibiotics by prohibiting pharmacies from selling antibiotics without prescriptions, which was widespread before the ruling [24]. Since our study has samples ranging over years 2014-2018, it has the potential of being the basis of future studies regarding the change of antibiotics resistance before and after the decision. One area to further strengthen our research is to obtain patient medical information to see if there are any comorbidities and by analyzing that information, we can add to our research the factors that may affect the morbidity and mortality of our patients.

## Conclusions

In conclusion, we had set out to find the prevalence of antimicrobial drug resistance and the susceptibility of *K. pneumoniae* at King Fahad Hospital, Medina, between January 1, 2014, and December 31, 2018. The research showed increased resistance rates to beta-lactams, as well as an emergence of resistance to carbapenems and other alternatives of treatment (colistin and tigecycline). In addition, cross-resistance rates with imipenem were high, with the lowest being colistin and tigecycline. Based on the findings, we can state that local surveillance of antibiotic-resistant organisms and tracking emerging resistance, as well as proper antibiotic use, are important in controlling the antibiotic resistance of *K. pneumoniae *as well as other organisms.
